# The R219K Polymorphism of the ATP Binding Cassette Subfamily a Member 1 Gene and Susceptibility to Ischemic Stroke in Chinese Population

**DOI:** 10.1515/med-2020-0039

**Published:** 2020-04-06

**Authors:** Jianmin Li, Ming Wen, Zhiping Zhang, Zhihua Qiu, Yiming Sun

**Affiliations:** 1 Department of Neurosurgery, Karamay Central Hospital of Xinjiang, Karamay City, Xinjiang Province, China, 834000; 2 Department of Pharmacy, Karamay Central Hospital of Xinjiang, MM, Karamay City, Xinjiang Province, China, 834000; 3 Department of Neurosurgery, Wuhan Wuchang Hospital, MM, Wuhan City, Hubei Province, China, 430063; 4 Department of Neurosurgery, Xiangya Hospital of Central South University, MD, Changsha City, Hunan Province, China, 410008; 5 Department of Neurosurgery, Zhuzhou Central Hospital, Zhuzhou City, Hunan Province, China, 412007

**Keywords:** ABCA1, Ischemic stroke, R219K, meta-analysis

## Abstract

Stroke is the major cause of death and disability worldwide. ABCA1 R219K has been suggested as a risk factor for ischemic stroke, but the results remain inconclusive in the Chinese population. This study aimed to assess the association between ABCA1 R219K and ischemic stroke using meta-analysis. A systematic literature search was conducted to select eligible studies and the pooled odds ratio (OR) with 95% confidence interval (CI) was used to evaluate the strength of association. Fourteen studies containing 2865 cases and 3227 controls were included in the meta-analysis and the results suggested that there is a strong association between ABCA1 R219K and the ischemic stroke risks (K vs. R: OR = 0.837, 95% CI: 0.735- 0.954, p=0.008; KK vs. RR: OR = 0.689, 95% CI: 0.520-0.912, p=0.009; KK+RK vs. RR: OR = 0.782, 95% CI: 0.691-0.885, p<0.001). Subgroup analysis revealed that significant association was found for the 4 genetic models (p<0.05) in the Southern population, while in the northern population significant association was only found under the dominant model (KK+RK vs. RR: OR = 0.744, 95% CI: 0.583- 0.949, p<0.017). This meta-analysis suggested that ABCA1 R219K polymorphism might be a protective factor against developing IS, indicating this SNP may contribute to the pathogenesis of ischemic stroke and might be potentially used as a biomarker to predict the susceptibility to ischemic stroke.

## 1 Introduction

Stroke is a common but serious cerebral vascular condition, and it is estimated to cause 5% of disability and 10% of deaths worldwide [1]. The lifetime risk of stroke is now 25% from the age of 25 years globally. Ischemic stroke (IS) is the main type of stroke, which accounts for more than 70% of the stroke [2]. In particular, the highest estimated lifetime risk of stroke is found in East Asia and it has become the leading cause of mortality in China [3]. Although both the environmental and genetic factors contribute to the pathogenesis of IS, the precise etiology of IS has not been completely elucidated yet [4]. Several genome-wide association studies (GWAS) and large collaborative efforts have been devoted to explore the genetic risk factors of IS, and many genetic variants such as *PITX2* and *ZFHX3* have been identified [5]. However, these genes only account for a portion of the overall genetic risk and the genetic risk factors may vary among populations. More studies on the genetic factors of IS, in particular, in individual populations are still required.

It is well accepted that the serum high-density lipo-protein cholesterol (HDL-C) level is an independent risk factor of vascular disease with the increased HDL-C providing a protective effect against vascular disease incidence. ATP binding cassette subfamily A member 1(ABCA1) is a key regulator of cholesterol efflux and plays a pivotal role in the synthesis of HDL-C and reverse cholesterol transport (RCT) [6]. The ABCA1 gene is located at chromosome 9q31.1, which encompasses 50 exons and encodes a product containing 2261 amino acids. A recent meta-analysis has shown that ABCA1 gene variation is associated with elevated blood lipid levels, especially the HDL-C [7]. Genetic polymorphisms within ABCA1 have been identified as the molecular basis of Tangier disease and familial hypoalphalipoproteinemia [8, 9]. Additionally, ABCA1 gene mutations have also been associated with decreased risk factors for coronary artery disease (CAD), another common vascular condition [10]. Hence, it was reasonable to hypothesize that gene polymorphisms in ABCA1 might affect the risks of IS.

R219K in exon 7, also known as rs2230806, is a common variation of the ABCA1 gene. The G to A substitution causes the change from Arg to Lysine at 219 site of the peptide. Although the impacts of protein function caused by this mutation is not clear, genetic studies have found that R219K variation is associated with either increased HDL-C and/or decreased risk of CAD [11]. Another study found that the ABCA1 R219K cloud modulate the association between HDL-C and age in Caucasians [12]. However, whether ABCA1 R219K is associated with the risks of IS has not been determined. In Caucasians, studies on the association between ABCA1 R219K and IS generated inconclusive results. Andrikovics and colleagues found a protective role of ABCA1 R219K in stroke in Hungarian patients [10]. However, Pasdar and colleagues found marginal difference in the ABCA1 R219K allele frequency in case and controls [13].

Abundant studies have also been performed to investigate such an association in the Chinese population. Xiao and colleagues first reported the ABCA1 R219K might be a protective factor for IS in a Southern Chinese population [14]. Notably, these findings were further confirmed by subsequent studies [15, 16], while others found ABCA1 R219K might increase the IS risks or lack association [17, 18]. The controversial results of associations between ABCA1 R219K and IS susceptibility may due to the relatively small sample size in the individual studies. Meta-analysis is a powerful tool to combine the results from individual studies and increase the power of obtaining a more precise conclusion. However, the meta-analysis of multiple populations may be affected by different genetic background. In order to reduce effects of these confounding factors, we focus our attention on the association between ABCA1 R219K and IS in the Chinese population and also perform the subgroup analysis based on the region of the population.

## 2 Methods

### 2.1 Literature search and inclusion criteria

The relevant literature, published before August 2019, was searched across the electronic database of Pubmed, WangFang and China National Knowledge Infrastructure (CNKI) in English or Chinese. The following key terms were used for the literature searching: “ischemic stroke”, “cerebrovascular accident”, “cerebrovascular disease”, “cerebral infarction”, “polymorphism”, “gene mutation” and “ABCA1”. A manual search was also carried out on the references of the literature to identify additional eligible studies.

The following criteria was used to select eligible studies for the meta-analysis: (1) studies evaluating the association between ABCA1 and ischemic stroke; (2) studies containing data from the Chinese population; (3) Clear diagnosis of ischemic stroke patients; (4) R219K polymorphism was genotyped and detailed frequency data available. Accordingly, studies that did not meet the above criteria were excluded. If there was more than one case-control study reported, they were treated independently.

### 2.2 Data extraction

Two authors performed the data extraction independently and any disagreements were resolved by discussion with a third author. Following information was extracted from the included studies: the surname of the first author, year of publication, region of the study (South or North China), numbers of controls and cases, genotype methods and distribution of alleles and genotypes. Additionally, P values of Hardy-Weinberg equilibrium (HWE) test for the controls were also extracted or calculated based on the genotype data.

### 2.3 Statistical analysis

The meta-analysis was performed as previously reported [19]. The analysis was carried out using STATA statistical software (Version 12.0; Stata Corporation, College Station, TX, USA). The pooled odds ratios (ORs) with 95% confidence intervals (CIs) were employed to estimate the association between R219K and ischemic stroke risk. During the ORs calculation, four genetic models were used: additive model (K vs. R), recessive model (KK vs. RK+RR), homozygotes model (KK vs. RR), and dominant model (KK + RK versus RR). A random model was used in the pooled ORs calculation. The significance of pooled ORs was evaluated by the Z test and a p<0.05 (two-tailed) was considered statistically significant. The potential heterogeneity among studies was assessed by the I^2^ test and p<0.1 for the I^2^ was considered statistically significant for the heterogeneity test. The Begg’s and Egger’s test were employed to test the potential publication bias and p<0.05 indicated significant publication bias. In addition, the funnel plot was also drawn based on the Begg’s test. Subgroup meta-analysis was also performed based on the region of population and Hardy–Weinberg equilibrium (HWE) test.

## 3 Results

### 3.1 Characteristics of included studies

**Figure 1:**
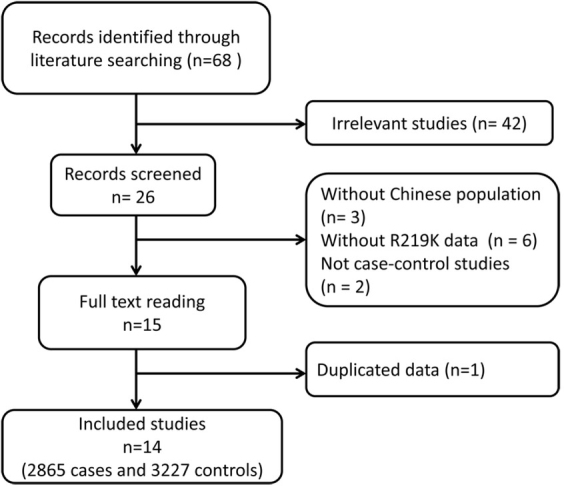
The flow diagram of the selection process for the meta-analysis

Through literature searching, 68 publications relevant to ABCA1 gene and ischemic stroke were identified. Follow-up title and/or abstract reading excluded 42 studies as they were irrelevant to the meta-analysis. After further abstract reading, 11 studies were excluded because there was no data from Chinese population (n=3), no R219K data, and not case control studies (n=2). Subsequently, full texts readings were performed on the 15 studies and 1 study was further excluded due to duplicated data. Finally, 14 studies, containing 2865 cases and 3227 controls, investigating the PD-L1 R219K C>G and the risks of ischemic stroke were included in the present meta-analysis (Fig. 1) [14, 15, 16, 17, 18, 20, 21, 22, 23, 24, 25, 26, 27, 28]. As shown in Table 1, the publication year varied from 2014 to 2015, and the genotype distributions in the controls were in agreement with HWE, except for one study.

**Table 1:** Characteristics of included studies for the meta-analysis of R219K and ischemic stroke in Chinese population

		Case				Control						
					
Study	Region	N	RR	RK	KK	N	RR	RK	KK	Genotype	HWE(P)	Reference
Xiao 2004	Hunan-South	379	149	172	58	351	112	172	67	PCR-RFLP	0.9467478	[14]
Cui 2005	Xi’An-North	96	15	35	46	90	21	45	24	PCR-RFLP	0.9915804	[17]
Wang 2007a	Xinjiang-North	58	19	35	4	60	14	36	10	PCR-RFLP	0.1116795	[18]
Wang 2007b	Xinjiang-North	58	23	27	8	60	21	34	5	PCR-RFLP	0.0882217	[18]
Deng 2008	Hunan-South	109	30	60	19	339	110	168	61	PCR-RFLP	0.8208377	[20]
Zhang 2008	Ningxia-North	177	43	87	47	234	40	129	65	PCR-RFLP	0.0777884	[21]
Liu 2009	Guangxi-South	131	70	52	9	135	53	60	22	PCR-RFLP	0.4739363	[15]
Zhao 2010	Hunan-South	211	69	94	48	211	68	111	32	PCR-RFLP	0.2241758	[22]
Wang 2010	Fujian-South	324	107	172	45	152	41	77	34	PCR-RFLP	0.8502736	[23]
Yi 2011	Guizhou-South	240	45	109	86	240	36	97	107	PCR-RFLP	0.0770234	[24]
Xue 2012	Fujian-South	182	70	91	24	229	62	118	49	PCR-RFLP	0.6079555	[16]
Zhang 2012	Ningxia-North	105	30	63	12	257	63	125	69	PCR-RFLP	0.6685466	[28]
Zhou 2013	Hunan-South	279	98	128	53	351	112	172	67	PCR-RFLP	0.9467478	[25]
Cai 2014	Hunan-South	156	65	48	43	160	65	45	50	PCR-RFLP	<0.0001	[26]
Sun 2015	Shandong-North	360	135	181	44	358	98	169	91	PCR-RFLP	0.2936262	[27]

### 3.2 Meta-analysis and heterogeneity test

**Figure 2:**
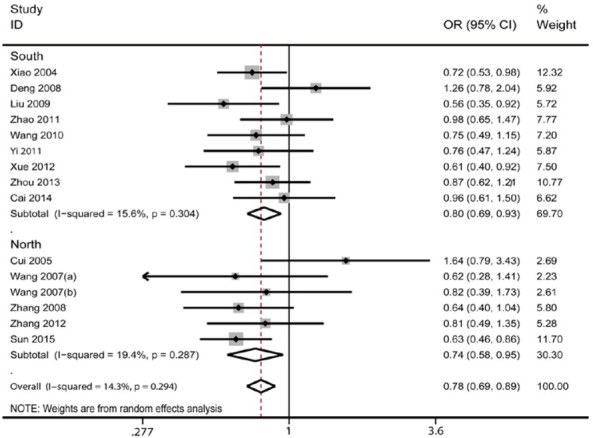
Forest plot of ORs for the association between ABCA1 R219K and ischemic stroke risk under the dominant model (KK+RK vs. RR, stratified by population region). The circle and horizontal lines represent the OR and 95 % CI and the area of the squares reflect the weight of individual studies included in the meta-analysis. The diamonds represent the pooled ORs and 95 % CI.

As shown in Table 2, ABCA1 R219K was significantly associated with ischemic stroke for the allelic model (K vs. R: OR = 0.837, 95% CI: 0.735- 0.954, p=0.008), the homozygotic model (KK vs. RR: OR = 0.689, 95% CI: 0.520-0.912, p=0.009) and the dominant model (KK+RK vs. RR: OR = 0.782, 95% CI: 0.691-0.885, p<0.001) (Fig. 2), but not the recessive model (KK vs. RK+RR: OR =0.772, 95% CI: 0.594-1.003, p=0.053). As the control population of one study deviated from HWE, this study was omitted in the further analysis but there was still significant association between ABCA1 R219K with ischemic stroke for 3 genetic models (p<005). P values from I2 test were used to detect the potential heterogeneity in the meta-analysis. Heterogeneity were found for the allelic model, the homozygotic model and the recessive model (p<0.001) but not for the dominant model (p<0.294). These results indicate that allele K might be a protective factor for ischemic stroke, but heterogeneity exist in the included populations.

**Table 2:** Meta-analysis on the association between ABCA1 R219K and ischemic stroke

					Publication bias (P)	
Population	Genetic model	Pooled OR (95% CI)	P	Heterogeneity (P)	Begg’s	Egger’s
	K vs. R	0.837 (0.735, 0.954)	0.008	0.000	0.784	0.495
	KK vs. RK+RR	0.772 (0.594, 1.003)	0.053	0.000	0.784	0.915
Overall	KK vs. RR	0.689 (0.520, 0.912)	0.009	0.000	0.927	0.724
	KK+RK vs. RR	0.782 (0.691, 0.885)	0.000	0.294	0.649	0.170

	K vs. R	0.838 (0.734, 0.957)	0.009	0.031	0.297	0.839
	KK vs. RK+RR	0.786 (0.619, 0.996)	0.046	0.025	1.000	0.797
South	KK vs. RR	0.718 (0.544, 0.949)	0.020	0.021	0.532	0.754
	KK+RK vs. RR	0.803 (0.693, 0.932)	0.004	0.304	1.000	0.640

	K vs. R	0.856 (0.632, 1.158)	0.313	0.000	0.188	0.304
	KK vs. RK+RR	0.773 (0.396, 1.510)	0.451	0.000	0.573	0.614
North	KK vs. RR	0.666 (0.342, 1.296)	0.232	0.000	0.348	0.423
	KK+RK vs. RR	0.744 (0.583, 0.949)	0.017	0.287	0.348	0.258

	K vs. R	0.833 (0.725, 0.957)	0.010	0.000	0.288	0.220
HWE	KK vs. RK+RR	0.767 (0.578, 1.017)	0.066	0.000	0.757	0.884
	KK vs. RR	0.677 (0.500, 0.915)	0.011	0.000	0.918	0.686
	KK+RK vs. RR	0.771 (0.677, 0.878)	0.000	0.280	0.757	0.193

By considering that genetic background between Southern and Northern Chinese population might be different, stratified analysis was conducted based on the region of the study. In the Southern population, significant association was found for the 4 genetic models (p<0.05); in the northern population, significant association was found under the dominant model (KK+RK vs. RR: OR = 0.744, 95% CI: 0.583- 0.949, p<0.017), but not under the allelic model, homozygotic model and recessive model (p>0.05). However, heterogeneity were still found in the allelic model, the homozygotic model and the recessive model (p<0.05) in both Southern and Northern populations. Taking together, the meta-analysis revealed that ABCA1 KK+RK carriers might have decreased risk of ischemic stroke in Chinese populations.

### 3.3 Publication bias

**Figure 3:**
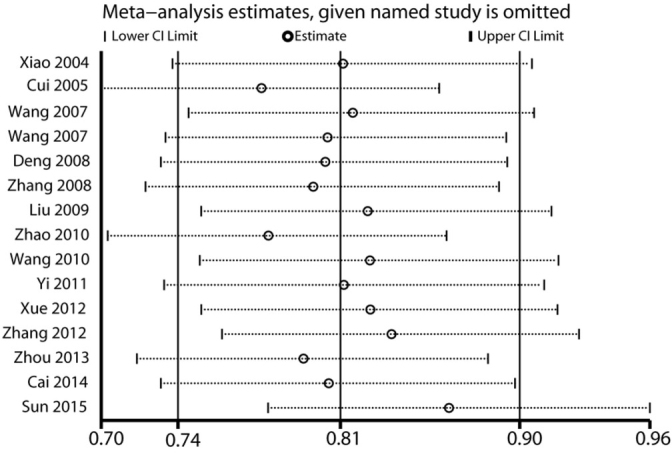
Sensitivity analysis for the association between ABCA1 R219K and ischemic stroke for the included studies.

Begg’s funnel plots and Egger’s tests were employed to evaluate the potential publication bias. The results of Begg’s and Egger’s tests are shown in Table 2 and a funnel plot under the dominant model (Fig. 3). Studies in the funnel plots were symmetrically distributed in the overall meta-analysis under all genetic models (p>0.05), suggesting the absence of publication bias for the meta-analysis of ischemic stroke risks.

### 3.4 Sensitivity Analysis

**Figure 4:**
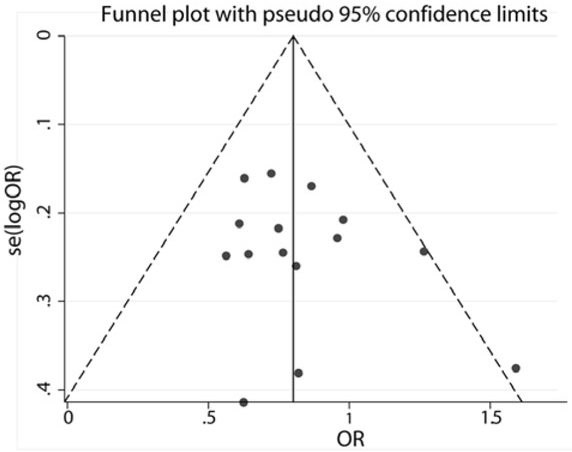
Begg’s funnel plot for association between ABCA1 R219K and ischemic stroke.

Sensitivity analysis was performed by replicating the analysis after omitting one study at a time to evaluate the effect of quality of studies on the final findings. A representative picture for the dominant model is shown in Fig. 4. The results found that the meta-analysis of the correlation between the ABCA1 R219K and ischemic stroke susceptibility remained unchanged in all genetic models.

## 4 Discussion

ABCA1 plays an important role in the development of IS. ABCA1 is a major regulator of cellular and systemic cholesterol homeostasis. It is widely expressed and contributes to reverse cholesterol transport (RCT) by exporting cholesterol out of cells to extracellular acceptors to form HDL-C. Through the above mentioned RCT, ABCA1 exerts its protective role in atherosclerosis (AS), which is believed to be an initial step in the IS pathogenesis. R219K is a common variation in the ABCA1 gene. Several studies investigated the association between ABCA1 R219K and IS susceptibility in Chinese population, but the results are inconsistent. The present meta-analysis included 14 eligible original studies containing the data of 2865 cases and 3227 controls. The main findings of this meta-analysis are that the allele K might be a protective factor for the IS and the ABCA1 KK+RK carriers might have decreased risk of ischemic stroke in Chinese populations. Subgroup meta-analysis shows a stronger association between ABCA1 R219K and IS in the Southern Chinese population than the Northern Chinese populations.

Hou and colleagues conducted a meta-analysis on the association between ABCA1 R219K and IS in the Chinese population in 2014 [29]. This study included 1619 IS cases and 1907 controls of 9 publications. The results showed that ABCA1 R219K was associated with IS in all genetic models except the recessive genetic model. However, five years have passed and more studies on the Chinese populations have been published. Through the literature search, 5 new publications were identified [22, 24, 25, 26, 27]. The numbers of cases and controls increased to 2865 and 3227 respectively in the present meta-analysis. We also found that the allele K of ABCA1 R219K might be a protective factor for IS and, particularly, the KK+RK carriers might have decreased risk of ischemic stroke in Chinese populations. Our results provided more convincing evidence for a protective role of ABCA1 R219Kin IS in a larger Chinese population.

More recently, a meta-analysis was carried out in the Asian and Caucasian populations and have found that homozygous RR of R219K was significantly associated with increased IS risk (OR = 1.31, 95% CI: 1.16-1.48; p<0.001) [30], which is consistent with our finding that ABCA1 KK+RK carriers might have decreased risk of ischemic stroke. However, the subgroup analysis revealed that such association was presented in Asian populations, but not in Caucasian populations, suggesting the heterogeneity among the populations may affect the association between ABCA1 gene polymorphism and IS. In the present meta-analysis, a modest difference was found between the Southern and the Northern Chinese population, indicating the potential heterogeneity may also existed in Asian groups. In addition, only two Caucasian studies were identified and included in the meta-analysis of Au and colleagues, such lack of association between ABCA1 R219K and IS may also be due to the limited study numbers. More studies are needed to clarify whether there is an association between ABCA1 R219K and IS in Caucasians.

Another question is how to explain the protective role of ABCA1 R219K in IS risk in the Chinese population. It is generally accepted that HDL-C is a protective factor against vascular disease . As a kind of cerebral vascular condition, increased serum HDL-C has been linked to decreased IS risk. Recent studies found that ABCA1 gene polymorphisms are associated with elevated blood lipid levels, in particular, the serum HDL-C levels. Considering the important regulatory role of ABCA1 in cholesterol homeostasis, it is likely that the protective role of ABCA1 R219K in IS is due to the elevated HDL-C. However, how the R219K variation affects the function of ABCA1 protein is unclear, and requires much more investigation in the future.

The present meta-analysis should be interpreted with caution due to several limitations. First, we only focused on R219K variation in the ABCA1 gene, while not evaluating other genes or environmental factors. It is possible that the potential roles of ABCA1 R219K are diluted or masked by other gene-gene or gene-environment interactions. Second, we only conducted the meta-analysis in the Chinese population, and so far only 2 studies have been published in Caucasians populations. Whether there is such association in Caucasian populations merits further investigations. The last but not the least, heterogeneity still exists even if we perform subgroup analysis based on the region of the Chinese populations. The association between ABCA1 R219K and IS should considered with caution when applied to a specific population.

## 5 Conclusion

This meta-analysis suggests that ABCA1 R219K polymorphism might be a protective factor against developing IS. However, the strength of association might vary among populations, and larger and well-designed studies are warranted to validate our findings.
